# World’s Columbian Dental Congress—Women Dentists

**Published:** 1893-05

**Authors:** Vida A. Latham

**Affiliations:** 96 State St., Chicago, Ill.


### NOTICES.

### World's Columbian Dental Congress—Women Dentists.

Finding a good deal of misunderstanding about the World's Fair Dental Congress, I therefore venture to give briefly an outline of it as far as it affects women dentists.

The World's Columbian Dental Congress meets August 14th to 19th, 1893, inclusive, and consists of (*a*) the General Dental Congress and its Subdivisions; (*b*) World's Congress Auxiliary, Dr. John S. Marshall, Chairman; (*c*) Woman's Branch of Auxiliary, Dr. Hattie E. Lawrence, Chairman.

Arrangements are completed which secure woman's entrance and participation in the Congress on exactly *the same terms as the men*, thus forming one united Congress.

Women are on the essay programmes and also give clinical demonstrations in operative and prosthetic dentistry.

Arrangements have been made to secure special headquarters in the Art Palace where the Dental Congress is held, and where the officers of the Woman's Committee will be pleased to give any information concerning the Dental Congress to all women dentists and their friends. Reports, current literature, etc., will be placed there for the convenience of members.

The essays, reports and all questions of interest will be printed in the proceedings of the Congress after having been accepted by the Committee.

A reception will be given, when the ladies can meet each other and discuss various matters of interest.

Among those who will be present, read papers, give clinics, etc., from home and abroad are: Doctors Olga Neymann, New York; Alice Ireland, New York; A. L. Cuinet, Brooklyn, N. Y.; Mary H. Stilwell, Philadelphia; Annie T. Focht, Philadelphia; Hannah Mercer Miller, Philadelphia; Eliza Yerkes, Philadelphia; Helen D. Searle, Kansas; May Weston, Kansas; Lucy Hobbs Taylor, Kansas; Julia A. Otte, Kansas; Olinda A. Otte, Kansas; Jessie Ritchie, Iowa; Emma Chase, St. Louis, Mo.; Luella Cool, California; —. Nehls, Michigan; Lucy K. Waterloo, Michigan; Alice L. Sherman-Barber, Wisconsin; Theckla Stein Reuter, Wisconsin; Clara McNaughton, D. C.; —. Jewell, D. C.; Fannie Hoopes, Baltimore; Hirschfeld-Tiburtius, Berlin; Emma Lacey, London; Olga Von Oetzen, England; Harrie Brierley-Bosworth, England; Dagmar Koldrup, Copenhagen, and others.

It may be interesting to know that the Women's Auxiliary Committee have the names and addresses of over 400 women dentists scattered all over the world.

The Woman's Dental Association of the United States, with Dr. Mary H. Stilwell as President, has arranged to hold a session during the week of the Congress, to which all women dentists are most cordially invited.

It must be distinctly understood that *there is only one World's Fair Columbian Dental Congress in* 1893 *in the United States* and it consists of *women and men* the latter having very cordially extended their help to place women on equality with themselves whenever they prove willing and capable.

The most cordial professional feeling exists between the sexes and all hope to have an excellent practical meeting and pleasant memories of the World's Columbian Dental Congress in Chicago, 1893, and, therefore it is the duty of every woman to be present.

Communications in the matter of papers, clinics, exhibits, photographs, will be gladly received.

A postal address with name and address to rectify and complete our Woman's Dental Directory and promise of attendance is most earnestly solicited at once, and may be addressed to Dr. Hattie E. Lawrence, Chairman, or to the undersigned, who will see that they are placed in the hands of the proper Committee.

DR. VIDA A. LATHAM,
Secretary, Pro. Tem.,
96 State St., Chicago, Ill.

